# The value of lactate dehydrogenase to albumin ratio and immune inflammation biomarkers in colorectal cancer

**DOI:** 10.3389/fsurg.2023.1118403

**Published:** 2023-03-01

**Authors:** Jiali Wu, Ao Wu, Songzi Wang, Chunxian Zeng, Ruizhi Wang, Juan Zhou, Dong Wang

**Affiliations:** ^1^ Department of Laboratory Medicine, The First Affiliated Hospital, Sun Yat-sen University, Guangzhou, China; ^2^ School of Cyber Science and Engineering, Southeast University, Nanjing, China; ^3^ Department of Gastroenterology, Shenzhen Hospital, Southern Medical University, Guangzhou, China; ^4^ Shenzhen Key Laboratory of Viral Oncology, Clinical Innovation & Research Center (CIRC), Shenzhen Hospital, Southern Medical University, Shenzhen, China; ^5^ Department of Oncology, General Hospital of the Southern Theatre Command, PLA, Guangzhou, China

**Keywords:** colorectal cancer, prognosis, marker, lactate dehydrogenase to albumin ratio, immune, inflammation

## Abstract

**Background:**

Colorectal cancer (CRC) is one of the most prevalent gastrointestinal cancers. Evidence for the importance of inflammation and immunology in the development and progression of CRC is growing steadily. The purpose of this study was to determine the clinical importance of Lactic Dehydrogenase (LDH) to Albumin (ALB) Ratio (LAR) and immune-inflammation biomarkers (IIBs) in patients with CRC.

**Methods:**

This study enrolled 382 CRC patients. The LAR was determined as the serum LDH(U/l) to ALB(g/l) ratio. We compared the levels of LAR and IIBs in different TNM stages and tumor differentiation. The relationship between LAR and IIBs and overall survival (OS) of CRC was determined by Cox regression models. A prognostic nomogram was created using the results of the multivariate analysis and the effectiveness of the nomogram was assessed using the ROC, calibration, and decision curves. We evaluated the relationship between LAR and IIBs and clinical features of CRC.

**Results:**

The levels of LAR, SII, NLR and PLR in TNM IV stage group (LAR:5.92 (5.23–8.24); SII: 1040.02 (499.51–1683.54); NLR: 2.87 (2.07–5.3); PLR:187.08 (125.31–276.63)) were significantly higher than those in other groups. LAR and NLR showed no significant difference in different tumor differentiation groups, while SII and PLR in undifferentiated groups (SII:543.72 (372.63–1110.20); PLR: 147.06 (106.04–203.92)) were significantly higher than those in well and moderate groups (SII: 474.29 (323.75–716.01); PLR: 126.28 (104.31–167.88)). LAR (HR = 1.317, 95% CI = 1.019–1.454), TNM stage (HR = 2.895, 95% CI = 1.838–4.559), age (HR = 1.766, 95% CI = 1.069–2.922) and lymphocytes (HR = 0.663, 95% CI = 0.456–0.963) were predictors of OS. IIBs, including SII, NLR, and PLR are independent of OS. The LAR-based nomogram AUCs of 1-year, 3-year and 5-year survival probabilities in the training cohort were 0.86, 0.72, and 0.71, respectively, and the AUCs of the validation cohort were 0.85, 0.71, and 0.69 respectively. The LAR-based nomogram's ROC curves and calibration curves demonstrated higher OS discriminative performance. The decision curves demonstrated greater net benefit in the survival prediction.

**Conclusion:**

Preoperative LAR is a potential prognostic marker in CRC patients, while SII, NLR, and PLR are independent of OS. LAR was associated with tumor stage in CRC patients, but not with tumor differentiation.

## 1. Introduction

Colorectal cancer (CRC) is one of the most frequent gastrointestinal cancers globally. The morbidity and mortality rates of CRC are currently ranked third and second globally, respectively (1). CRC is the fifth most common reason for cancer-related death in China (2). Despite advancements in diagnostic methods, the majority of CRC patients still have intermediate and late-stage diagnosis. The 5-year survival for CRC has increased from 50% to 64% in the last 40 years due to early screening, advances in imaging and treatment (3). Studies have found that about one-third of patients who have received therapeutic surgery for CRC experience postoperative recurrence (4). More than half of the cases can reduce the incidence by adjusting risk factors, and the mortality can be reduced through correct screening and follow-up. Early diagnosis may lead to a higher cure rate, and effective monitoring after surgery can help identify the progression of the tumor and prompt treatment. Therefore, it is critical to find more economical and effective prognostic indicators to decide appropriate treatment regimens.

Systemic inflammation is considered as a marker of cancer and is associated with tumor development, metastasis and prognosis (5). Recent studies suggested that systemic inflammatory markers and biomarker combinations could be used as prognostic biomarkers for cancers. Preoperative examination results can help predict complications, and some preventive measures may be taken based on the examination results. IIBs are a group of markers that represent the host's inflammatory and immunological condition. IIBs, such as neutrophil/lymphocyte ratio (NLR), pan-immune-inflammation value (PIV), platelet/lymphocyte ratio (PLR), and systemic immune inflammation index (SII), are closely related to CRC (6–9). LDH to ALB ratio (LAR), a novel biomarker, has been demonstrated to be related to a bad prognosis in pancreatic and esophageal malignancies (10, 11). However, the significance of LAR in CRC patients in the Chinese population has been less reported. The latest results from Hu et al. (12) suggest that LAR could be a prognostic factor for OS and DFS. However, the relationship between LAR and clinical features was not explored. In this study, we investigated the correlation between IIBs and TNM stage and tumor differentiation in CRC patients, further verifying the role of LAR in CRC.

## 2. Methods

### 2.1. Characteristics of patients

**Figure 1:**
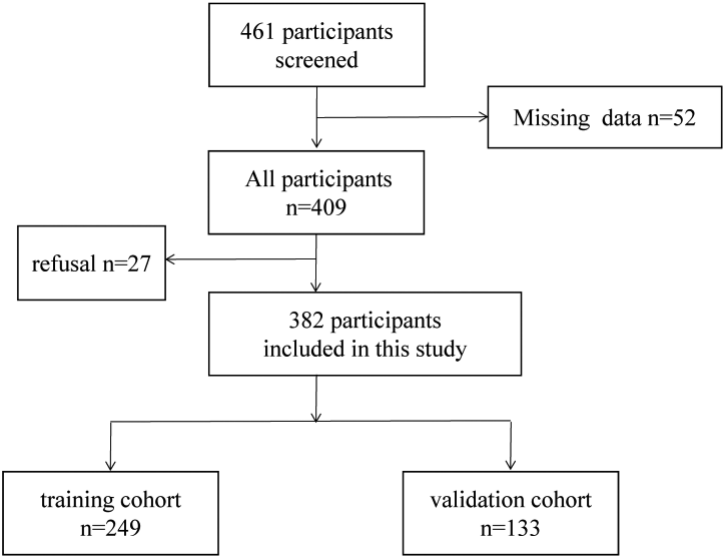
Flowchart of participants selection.

382 patients diagnosed as CRC were recruited from the First Affiliated Hospital of Sun Yat-sen University between October 2015 and November 2019. 382 CRC patients were randomly specified to the training cohort (*n* = 249) and the validation group (*n* = 133) (Figure 1). From the date of operation until the date of death or the final follow-up, overall survival (OS) was calculated. We followed up the CRC patients and the endpoints of this study were OS. According to the NCCN guideline (13) for CRC, all CRC patients were diagnosed as CRC by postoperative pathology or tissue biopsy. Meanwhile, the patients did not have other tumor diseases at the same time. The exclusion criteria are as follows: (1) pathology did not support the diagnosis of CRC; (2) recurrence of CRC; (3) received an anti-tumor therapy before resection; (4)suffering from infectious diseases and autoimmune diseases before resection; (5)incomplete clinical data.

This retrospective study was approved by the Human Research Ethics Committees of the First Affiliated Hospital of Sun Yat-sen University (approval number:[2021] No.299). Informed consent was obtained from all participants.

### 2.2. Laboratory measurements

Blood samples (3 ml) were collected from every participant in fasting state. Serum was separated by centrifugation at room temperature, 3000 g for 5 min. Routine blood test results and follow-up data were collected for each patient. The serum levels of CREA, ALT, AST, GGT, ALP, TP, ALB, TBIL and LDH were detected by a Beckman automatic biochemical analyzer (United States). Automatic blood cell analyzer (Sysmex XN-9000) was used to test. WBC, Neutrophils, Lymphocytes, RBC and PLT are tested by fluorescence staining and electrical resistance method. HGB, CREA, ALT, AST, GGT, ALP, TP, ALB, TBIL and LDH were detected by colorimetric method. All tests were performed at pH and temperature specified in the reagent specification. PLR = PLT count (10^12^/L)/lymphocyte count (10^9^/L); NLR = neutrophil count (10^9^/L)/lymphocyte count (10^9^/L); SII = [neutrophil count (10^9^/L) × platelet count (10^9^/L)]/lymphocyte count (10^9^/L).

### 2.3. Statistical analysis

Categorical data are shown as percentages, whereas continuous data are shown as median (range). To examine the independent prognostic risk variables for CRC, a Cox regression model was utilized. The multivariate cox regression analysis included significant components found in the univariate study. These analyses were performed with SPSS 23.0 (SPSS, United States). The nomogram for prognostic factors associated with OS was established with the rms package in R version 4.2.1 (http://www.r-project.org/). The performance of nomogram was evaluated by ROC curves and calibration curves. The improvement in survival prediction accuracy is measured by time-dependent receiver operating characteristic curves with areas under the curves (AUCs). The prognostic prognosis was more accurate the higher the AUC. The net benefit of the nomogram was assessed with the aid of decision curve analysis (DCA). Statistical significance was defined as *p *< 0.05.

## 3. Results

### 3.1. Characteristics of CRC patients

The clinicopathological characteristics of the training and validation cohorts were showed in Table 1. Of the 382 patients including 153 males and 96 females in the training cohort, 154 patients were diagnosed with TNM stage I + II, while 95 patients with TNM stage III + IV.

**Table 1:** Demographic and clinical characteristics of CRC patients.

Characteristics	Training cohort (*n* = 249)	Validation cohort (*n* = 133)	*P*
**Sex**			
Male	153 (61.4%)	78 (58.6%)	
Female	96 (38.6%)	55 (41.4%)	0.594
**Age(years)**			
≤60	109 (43.8%)	70 (52.6%)	
>60	140 (56.2%)	63 (47.4%)	0.098
**TNM stage**			
I + II	154 (61.8%)	67 (50.4%)	
III + IV	95 (38.2%)	66 (49.6%)	0.031
**Tumor differentiation**			
Well and moderate	215 (86.3%)	110 (82.7%)	
Undifferentiated	34 (13.7%)	23 (17.3%)	0.342
WBC(x10^9^/L)	6.55 (5.67–7.71)	6.395 (5.26–8.045)	0.371
Neutrophils (x10^9^/L)	3.83 (3.03–4.77)	3.685 (2.8–5.037)	0.817
Lymphocytes(x10^9^/L)	1.87 (1.52–2.31)	1.83 (1.458–2.175)	0.122
RBC(x10^12^/L)	4.49 (4.22–4.83)	4.56 (4.14–4.88)	<0.001
HCT	0.403 (0.355–0.434)	0.371 (0.310–0.405)	0.676
MCV(fL)	89.9 (84.2–92.8)	87.105 (81.118–91.175)	0.093
HGB(g/L)	130 (115–143)	123.5 (100.25–135)	<0.001
PLT (x10^9^/L)	251 (215–298)	243.5 (193–301.75)	0.437
CREA(mmol/L)	75 (62–88)	63.5 (52–76)	0.013
GGT(U/L)	20 (16–27)	20 (14–30)	0.741
ALB (g/L)	42.8 (39.2–45.9)	35.95 (31.4–39.875)	<0.001
ALT(U/L)	17.6 (13.1–23.8)	15 (11–20)	0.119
AST(U/L)	20.5 (17.4–24.9)	20 (17–26)	0.949
ALP (U/L)	71 (60–81)	73 (63–94.5)	0.022
LDH(U/L)	164 (148–190)	182 (163–217)	<0.001
TBIL(*μ*mol/L)	10.3 (7.4–13.9)	12 (9.25–16.05)	<0.001
TP (g/L)	71.8 (66.7–75.25)	70.9 (65.40–75.63)	<0.001
NLR	1.958 (1.478–2.549)	1.955 (1.550–2.816)	0.266
PLR	129.644 (104.762–171.97)	125.722 (103.349–185.608)	0.807
SII	474.946 (327.67–725.961)	479.121 (342.578–829.215)	0.404
LAR	3.832 (3.389–4.483)	5.147 (4.4–6.316)	<0.001

WBC, white blood cell; RBC, red blood cell; HCT, red blood cell specific volume; MCV, mean corpusular volume; HGB, hemoglobin; PLT, platelet count; BUN, Blood urea nitrogen; CREA, creatinine; GGT, gamma-glutamyl transpeptidase; ALB, albumin; ALT, alanine aminotransferase; AST, aspartate aminotransferase; ALP, alkaline phosphatase; LDH, lactate dehydrogenase; TBIL, total bilirubin; TP, total protein; NLR, neutrophil to lymphocyte ratio; PLR, platelet-to-lymphocyte ratio; SII, platelet counts ∗ neutrophil counts/lymphocyte counts; LAR, Lactate dehydrogenase to albumin ratio.

Among the 133 patients including 78 males and 55 females in the validation cohort, 67 patients were diagnosed with TNM stage I + II, while 66 patients with TNM stage III + IV. Overall, 86.3%, and 13.7% of the patients in the training cohort had well and moderate differentiation and undifferentiated differentiation, respectively, and 82.7% and 17.3% had well and moderate differentiation and undifferentiated differentiation, respectively in the validation cohort.

### 3.2. Relationship between IIBs and clinicopathological features in all CRC patients

**Figure 2:**
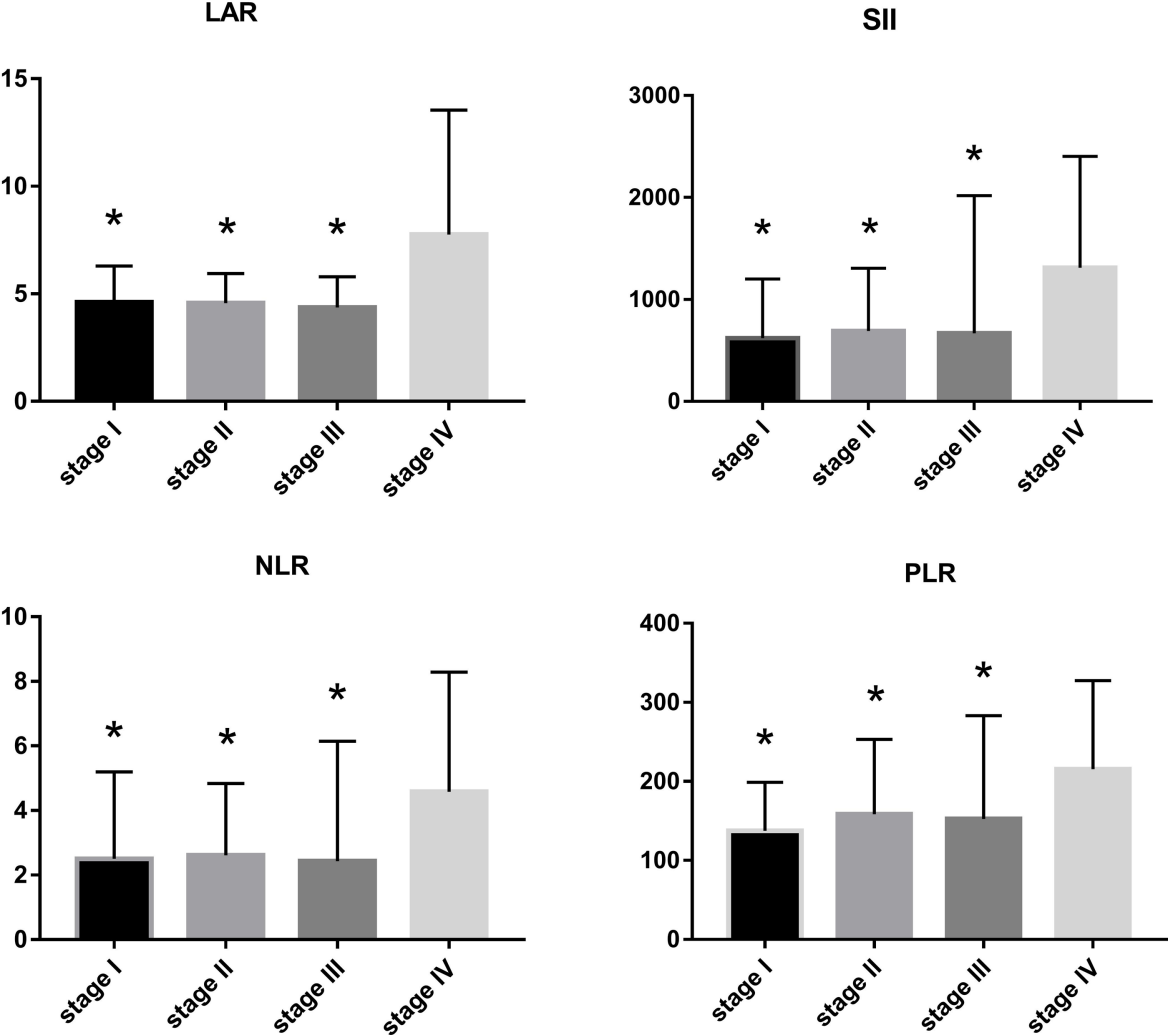
LAR, NLR, PLR and SII levels of CRC patients in different stages compared with stage IV, *p < 0.05, compared with the TNM stage IV group.

**Figure 3:**
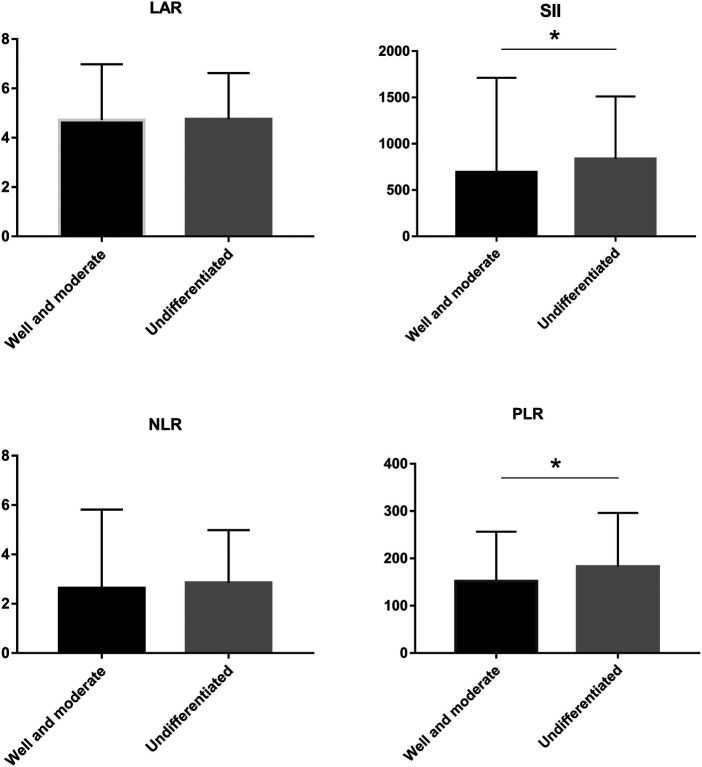
LAR, NLR, PLR and SII levels of CRC patients in different tumor differenation degree *p < 0.05.

LAR and IIBs levels at different TNM stage and tumor differentiation degree were observed in all CRC patients. The levels of LAR, SII, NLR and PLR in patients with stage IV were higher than those in patients with stage I, stage II and stage III (*p *< 0.05). The undifferentiated group had significantly higher SII and PLR levels (*p *< 0.05). There was no significant difference in LAR and NLR levels among all CRC patients in different tumor differentiation degree groups (*p* > 0.05) (Figures 2 and 3, Tables 2 and 3).

**Table 2:** Correlation between immune-inflammatory biomarkers (IIBs) and TNM stage in all CRC patients.

Variables	TNM stage				*p*
	I (*n* = 69)	II (*n* = 152)	III (*n* = 135)	IV (*n* = 26)	
LAR	4.1 (3.6–5.3)	4.23 (3.61–5.27)	4.01 (3.41–4.83)	5.92 (5.23–8.24)	<0.001
SII	447.44 (320.27–636.54)	466.09 (339.62–790.6)	474.95 (319.91–654.16)	1040.02 (499.51–1683.54)	<0.001
NLR	1.90 (1.51–2.4)	2.03 (1.46–2.95)	1.81 (1.5–2.42)	2.87 (2.07–5.3)	0.001
PLR	125.37 (104.95–151.16)	129.13 (104.13–181.59)	126 (103.21–171.89)	187.08 (125.31–276.63)	0.005

**Table 3:** Correlation between immune-inflammatory biomarkers (IIBs) and tumor differentiation in all CRC patients.

Variables	Tumor differentiation		*p*
	Well and moderate (*n* = 325)	Undifferentiated (*n* = 57)	
LAR	4.23 (3.60–5.21)	4.14 (3.48–5.30)	0.957
SII	474.29 (323.75–716.01)	543.72 (372.63–1110.20)	0.039
NLR	1.94 (1.5–2.55)	2.01 (1.56–3.5)	0.179
PLR	126.28 (104.31–167.88)	147.06 (106.04–203.92)	0.045

### 3.3. Cox regression analyses of prognostic factors for OS

Univariate analysis identified that TNM III + IV stage, age, lymphocytes, RBC,TP, HGB, CREA, ALB, NLR and LAR were significantly associated with poor survival. Multivariate analysis results showed that LAR (HR = 1.317, 95% CI = 1.019–1.454, *P* = 0.030) remained as an independent predictor for OS. Moreover, TNM stage (HR = 2.895, 95% CI = 1.838–4.559, *P *< 0.001), age (HR = 1.766, 95% CI = 1.069–2.922, *P* = 0.026) and lymphocytes (HR = 0.663, 95% CI = 0.456–0.963, *P* = 0.031) were also independent prognostic factors(Table 4). The results showed that SII, NLR and PLR were not associated with the prognosis of CRC patients in this study.

**Table 4:** Univariate and multivariate analyses of OS in training cohort.

Variables	Univariate analyses		Multivariate analyses	
	HR (95% CI)	*P*	HR (95% CI)	*P*
**Sex**				
Male	Reference			
Female	0.933 (0.592–1.470)	0.765		
**Age(years)**				
≤60	Reference			
>60	1.686 (1.054–2.696)	0.029	1.766 (1.069–2.922)	0.026
**TNM stage**				
I + II	Reference			
III + IV	2.495 (1.599–3.893)	<0.001	2.895 (1.838–4.559)	<0.001
**Tumor differentiation**				
Well and moderate	Reference			
Undifferentiated	1.47 (0.908–2.379)	0.117		
WBC(x10^9^/L)	0.900 (0.791–1.025)	0.113		
Neutrophils (x10^9^/L)	0.954 (0.833–1.092)	0.496		
Lymphocytes(x10^9^/L)	0.611 (0.417–0.897)	0.012	0.663 (0.456–0.963)	0.031
RBC(x10^12^/L)	0.670 (0.454–0.987)	0.043		
HCT	1.017 (0.996–1.039)	0.108		
MCV(fL)	0.990 (0.968–1.013)	0.399		
HGB (g/L)	0.990 (0.980–0.999)	0.038		
PLT(x10^9^/L)	0.998 (0.995–1.001)	0.228		
CREA (mmol/L)	1.007 (1.002–1.012)	0.011		
GGT(U/L)	0.999 (0.992–1.006)	0.745		
ALB (g/L)	0.948 (0.911–0.987)	0.009		
ALT(U/L)	1.002 (0.984–1.019)	0.864		
AST (U/L)	1.010 (0.987–1.034)	0.409		
ALP(U/L)	0.997 (0.986–1.008)	0.625		
LDH (U/L)	1.003 (0.997–1.009)	0.309		
TBIL(μmol/L)	0.975 (0.935–1.016)	0.228		
TP (g/L)	0.972 (0.944–1.000)	0.054		
NLR	1.041 (0.995–1.089)	0.084		
PLR	1.001 (1.000–1.003)	0.112		
SII	1.000 (1.000–1.000)	0.181		
LAR	1.277 (1.079–1.512)	0.004	1.127 (1.019–1.454)	0.030

OS, overall survival; HR, hazard ratio; The variables found significant at *P* < 0.1 in univariable analyses were entered into multivariable cox regression analyses.

### 3.4. Nomogram for OS establishment and validation

**Figure 4:**
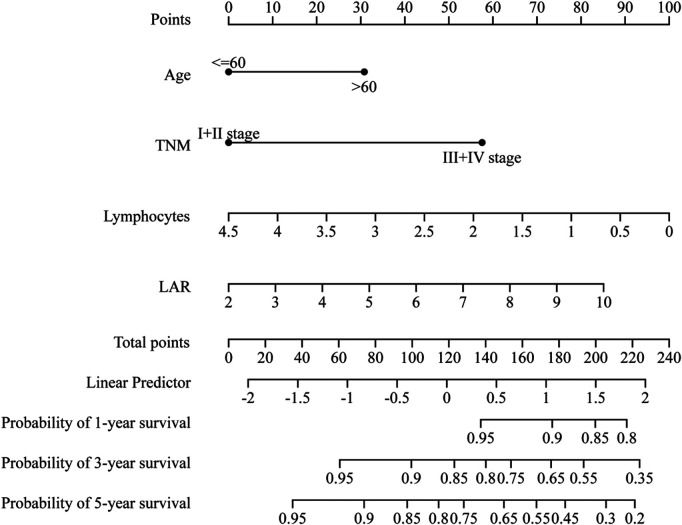
LAR-nomogram for predicting 1-year, 3- year and 5- year survival of CRC patients.

**Figure 5:**
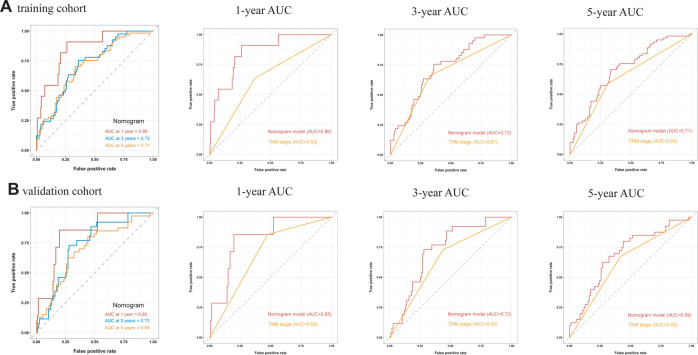
Time-independent ROC curves of the LAR-nomogram and TNM stage for 1-year, 3-year and 5-year survival prediction.

**Figure 6:**
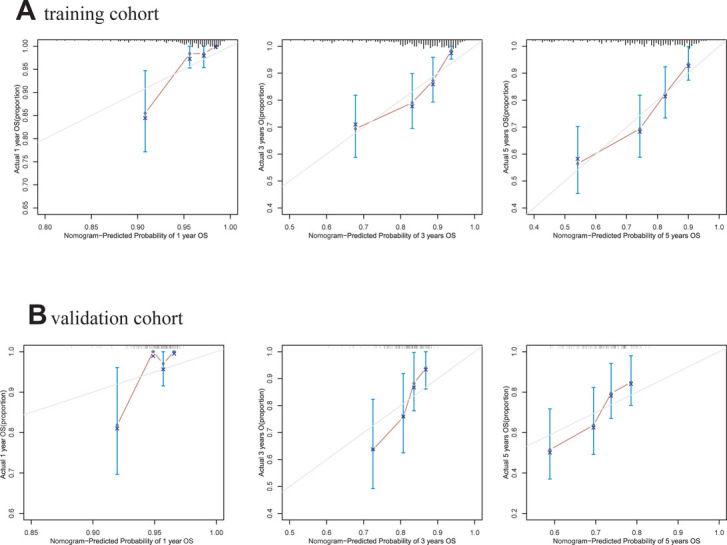
Calibration curves of the LAR-nomogram for 1-year, 3- years and 5- years survival prediction in the training and validation cohort. (**A**). training cohort. (**B**). validation cohort.

A comprehensive nomogram was developed to predict the survival rate of CRC patients, integrating the age, TNM stage, lymphocytes, and LAR (Figure 4). The LAR- nomogram's C-index was 0.69 (95% CI: 0.63- 0.74, *p* < 0.001). Using time-dependent ROC curves, the nomogram's prediction accuracy was assessed (Figure 5). In the training cohort, the AUCs of the prognostic nomogram of 1-year, 3-year and 5-year survival probability were 0.86 (95% CI: 0.75–0.96), 0.72 (95% CI: 0.64–0.80) and 0.71 (95% CI: 0.64–0.78), which were significantly higher than the TNM stage (1-year: AUC: 0.63, 95% CI: 0.51–0.74, 3-year: AUC: 0.67, 95% CI: 0.59–0.75 and 5-year: AUC: 0.64, 95% CI: 0.57–0.71). Calibration curves for 1-year, 3-years, and 5-years OS revealed strong relationships between nomogram calculated and real estimations (Figure 6).

**Figure 7:**
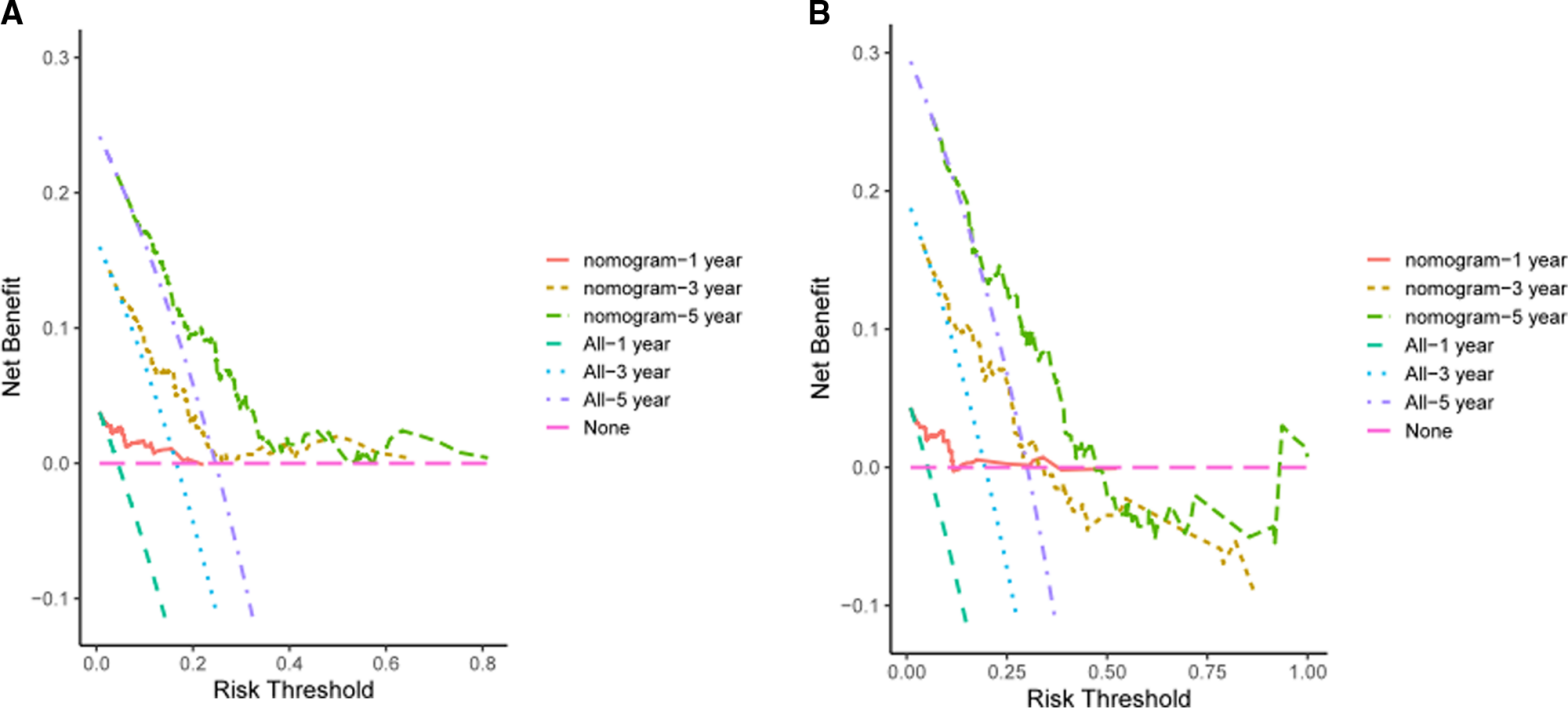
DCA of the LAR-nomogram for 1-year, 3-year and 5-year survival prediction in the training cohort (**A**) and validation cohort (**B**).

In the validation cohort, the AUCs of the prognostic nomogram of 1-year, 3-year and 5-year survival probability were 0.85 (95% CI: 0.70–0.99), 0.71 (95% CI: 0.60–0.82) and 0.69 (95% CI: 0.58–0.79), which were significantly higher than the TNM stage (1-year: AUC: 0.69, 95% CI: 0.54–0.84, 3-year: AUC: 0.65, 95% CI: 0.55–0.74 and 5-year: AUC: 0.63, 95% CI: 0.54–0.72). The clinical usefulness of our model was evaluated using decision curve analysis (DCA) curves (Figure 7). These results demonstrated that our approach had good practical application in estimating the 1-year, 3-year, and 5-year survival probabilities of CRC patients. Overall, the nomogram model demonstrated that it exceeded the TNM stage in terms of performance.

### 3.5. Relationship between LAR and clinicopathological features in all CRC patients

**Figure 8:**
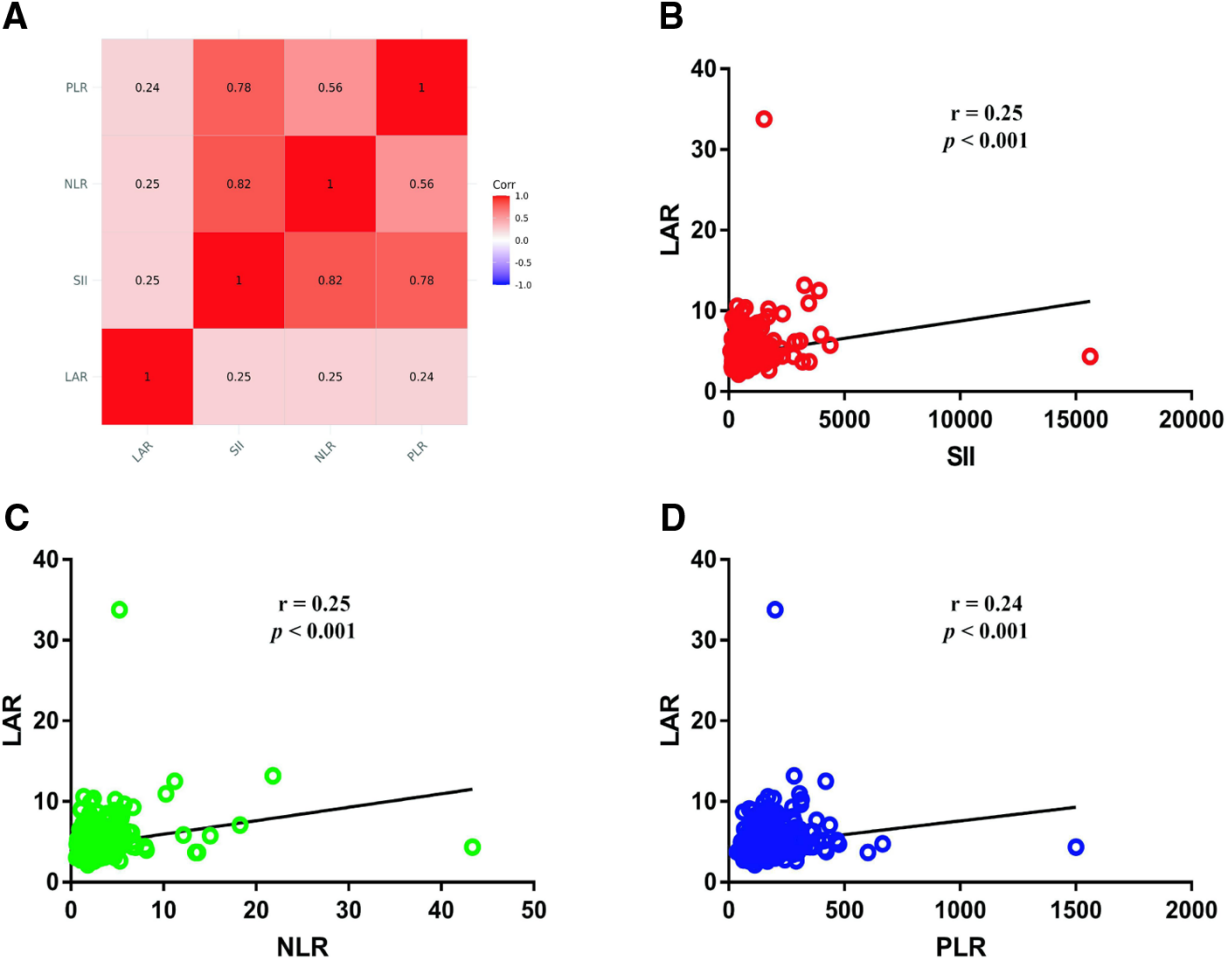
Correlation between LAR and SII, NLR and PLR (**A**). (**B**) Correlation between LAR and SII; (**C**) Correlation between LAR and NLR; (**D**) Correlation between LAR and PLR.

Patients were divided into the high LAR (> median LAR) group and the low LAR (< median LAR) group (Table 5). There were statistically significant differences in SII, NLR, PLR, T stage, M stage and TNM stage between patients with high LAR group and low LAR group (*p* < 0.05, Table 3). However, there were no differences in tumor differentiation and N-stage. CRC patients with high LAR were more likely to have high SII, NLR and PLR levels compared with those in the low LAR group. Spearman's rank correlation coefficient showed that LAR was positively correlated with SII (*r* = 0.25, *p* < 0.001), NLR (*r* = 0.25, *p* < 0.001) and PLR (*r* = 0.24, *p *< 0.001). In addition, the scatter diagram and thermogram show the correlation between LAR and the variables (Figure 8).

**Table 5:** Correlation between LAR and clinicopathological features in all CRC patients.

Variables	Low LAR (*n* = 191)	High LAR (*n* = 191)	*p*-value
Age (years)			<0.001
≤60	107 (56.0%)	72 (37.7%)	
>60	84 (44.0%)	119 (62.3%)	
LAR	3.575 (3.224–3.835)	5.256 (4.677–6.240)	<0.001
SII	424.125 (317.088–591.56)	570.957 (371.315–1023.167)	<0.001
NLR	1.792 (1.414–2.315)	2.133 (1.642–3.410)	<0.001
PLR	123.041 (97.649–151.298)	141.447 (108.520–202.864)	<0.001
Tumor differentiation			0.667
Well and moderate	161 (84.3%)	164 (85.9%)	
Undifferentiated	30 (15.7%)	27 (14.1%)	
T stage			<0.001
T1	21 (11.0%)	10 (5.2%)	
T2	27 (14.1%)	27 (14.1%)	
T3	130 (68.1%)	86 (45.0%)	
T4	13 (6.8%)	68 (35.6%)	
N stage			0.718
N0	112 (58.6%)	118 (61.8%)	
N1	54 (28.3%)	47 (24.6%)	
N2	25 (13.1%)	26 (13.6%)	
M stage			<0.001
M0	190 (99.5%)	167 (87.4%)	
M1	1 (0.5%)	24 (12.6%)	
TNM stage			<0.001
I	37 (19.4%)	32 (16.8%)	
II	75 (39.3%)	77 (40.3%)	
III	78 (40.8%)	57 (29.8%)	
IV	1 (0.5%)	25 (13.1%)	

## 4. Discussion

The recurrence and prognosis of CRC are closely related to the TNM stage. However, the prognosis of patients with the same stage and similar treatment regimens is not completely consistent (14). Numerous studies have demonstrated inflammatory cytokines can be used as prognostic markers in human malignancies (15, 16) and have shown that LAR and IIBs are associated with prognosis of tumors. Based on these considerations, this study was designed to explore the relationship between LAR and IIBs and CRC patients in a Chinese population. Our results indicated that LAR was a significant independent risk factor for OS. IIBs, including SII, NLR, and PLR are independent of OS. The TNM IV stage group had significantly higher LAR, SII, NLR and PLR levels. LAR and NLR showed no significant difference in different tumor differentiation groups, while SII and PLR in undifferentiated groups were significantly higher than those in well and moderate groups. T4 stage, M0 stage and TNM IV stage groups had higher LAR levels, suggesting that high levels of LAR are associated with poor outcomes in CRC patients.

Metabolic changes are one of the important features of tumors (17). The glucose metabolic reprogramming plays an important role in tumor genesis and development. In the tumor microenvironment, LDH is one of the key enzymes in the reprogramming of glucose metabolism and can catalyze the conversion of pyruvate to lactic acid. Studies have shown that even when oxygen levels are normal, tumor cells still metabolize glucose mainly through the glycolysis pathway, producing large amounts of lactic acid. The lactic acid effusion into the stroma of tumor cells makes the tumor microenvironment acidic (18), promotes tumor invasion, migration (19, 20) and immune escape (21, 22). Meanwhile, LDH can regulate tumor angiogenesis (23). LDH is expressed in many tissues. When cells are damaged, LDH is released into the bloodstream. Increased serum LDH levels due to tissue destruction by tumor growth and metastasis suggest that LDH may be a potential diagnostic marker for cancer. Studies have revealed that LDH and the prognosis of malignant tumors are tightly connected (24–26). Serum ALB level can reflect the nutritional status of the body and is related to tumor-related systemic inflammatory response. Studies have shown that malnutrition and inflammation can inhibit the synthesis of ALB and the ALB-related combinations are closely related to the prognosis of malignant tumors (27, 28). However, serum LDH and ALB levels may be affected by a variety of diseases, and combining LDH with ALB can reduce the disease-induced bias. Gao et al. showed that the pancreatic cancer patients of the higher LAR had significantly poorer OS (10). Feng et al. found the esophageal squamous cell carcinoma patients with LAR < 5.5 or less had a better 5 - year survival than the patients with LAR > 5.5 (11). Meanwhile, Ulas et al. reported that the high LAR is an independent prognostic factor for patients with OS in CRC, but did not provide a prognostic model for CRC (29). Therefore, this study developed a CRC prediction model and examined the connection between LAR and prognosis in CRC patients. The findings demonstrated that LAR is a significant independent risk factor for OS. The findings of Hu et al. (12) are the same as this. Additionally, contrary to the findings of several previous research, our investigation demonstrated that IIBs were not linked with OS of CRC. Feng et al. showed that SII was associated with postoperative infection complications and could predict the long-term prognosis of CRC patients (30). Satake et al. showed that PLR measurement after adjuvant therapy could predict the recurrence of CRC after surgical treatment (31). The inconsistencies in the findings may be related to population characteristics. The connection between IIBs and CRC has to be confirmed by larger investigations.

Some restrictions applied to our investigation. First, the results need to be further validated in multi-center studies with more patients because this was a retrospective, single-center investigation. Second, our study did not compare preoperative and postoperative changes in each index. Third, due to the limitations of the study, there may be some inevitable bias in our study, which may affect the results. Meanwhile, our results did not provide a cut-off threshold for clinical evaluation of patient outcomes. To confirm the predictive usefulness of LAR in CRC patients, further prospective studies are required.

In conclusion, our study identified LAR as a potential combination of biomarkers for the prognosis of patients with CRC. IIBs (SII, NLR and PLR) are not associated with the prognosis of CRC tumors, but with clinical features. High levels of LAR are associated with a poor prognosis for CRC. Preoperatively LAR can help physicians make more effective perioperative management and adjuvant treatment decisions after surgery.

## Data Availability

The original contributions presented in the study are included in the article/Supplementary material, further inquiries can be directed to the corresponding author/s.
